# Yes-Associated Protein 1: Role and Treatment Prospects in Orthopedic Degenerative Diseases

**DOI:** 10.3389/fcell.2020.573455

**Published:** 2020-10-15

**Authors:** Wenqing Xie, Wenfeng Xiao, Kun Tang, Liyang Zhang, Yusheng Li

**Affiliations:** ^1^Department of Orthopedics, Xiangya Hospital, Central South University, Changsha, China; ^2^National Clinical Research Center for Geriatric Disorders, Xiangya Hospital, Central South University, Changsha, China; ^3^Discipline Construction Office, Xiangya Hospital, Central South University, Changsha, China; ^4^Department of Neurosurgery, Xiangya Hospital, Central South University, Changsha, China

**Keywords:** yes-associated protein 1, osteoarthritis, osteoporosis, chondrocyte, mesenchymal stem cells

## Abstract

The Hippo/yes-associated protein 1 signaling pathway is an evolutionarily conserved signaling pathway. This signaling pathway is primarily involved in the regulation of stem cell self-renewal, organ size and tissue regeneration by regulating cell proliferation, differentiation and apoptosis. It plays an important role in embryonic development and tissue organ formation. Yes-associated protein 1 (YAP1) is a key transcription factor in the Hippo signaling pathway and is negatively regulated by this pathway. Changes in YAP1 expression levels affect the occurrence and development of a variety of tumors, but the specific mechanism associated with this phenomenon has not been thoroughly studied. Recently, several studies have described the role of YAP1 in osteoarthritis (OA). Indeed, YAP1 is involved in orthopedic degenerative diseases such as osteoporosis (OP) in addition to OA. In this review, we will summarize the significance of YAP1 in orthopedic degenerative diseases and discuss the potential of the targeted modulation of YAP1 for the treatment of these diseases.

## Introduction

YAP1, a connexin and transcriptional co-activator that plays an important role in cells, was first discovered by Sudol (1994) in 1994 and is the core factor of the Hippo signaling pathway (Huang et al., 2005; Dong et al., 2007). In mammalian cells, the primary members of Hippo/YAP1 signaling pathway include mammalian sterile 20-like kinase 1/2 (MST1/2), savlador family WW domain containing protein 1 (SAV1), large tumor suppressor homolog kinases 1/2 (LATS1/2), Mps one binder 1 (MOB1), YAP1, transcriptional co-activator with PDZ-binding motif (TAZ), and the TEAD family of transcription factors (TEAD1-4) (Kodaka and Hata, 2015). It is worth noting that YAP1 protein is regarded as the analog of TAZ. It has 488 amino acids, about half of the oxygen acid sequence of it are identical to TAZ and it has the same topological structure as TAZ (Low et al., 2014). The functions of YAP1 and TAZ are often redundant, which are generally referred to as YAP1/TAZ. However, YAP1 and TAZ can also function independently, and YAP1 has stronger influence than TAZ (Low et al., 2014; Plouffe et al., 2018). In terms of function, YAP1 and TAZ are mainly transcription co activators and multifunctional intracellular connexins, which participate in intracellular signal transduction and transcriptional co-activation of their downstream target factors (Hong and Guan, 2012). The Hippo/YAP1 signaling pathway exerts its functions through regulation of the above mentioned signaling pathway members. In a series of enzymatic cascade reactions that occur when the Hippo pathway is activated, MST1/2 first binds to SAV to undergo protein kinase phosphorylation, which then phosphorylates LATS1/2 and MOB1, and finally YAP1/TAZ effector factors are phosphorylated, allowing them to bind to intracytoplasmic 14-3-3 proteins and decompose together, forcing YAP1/TAZ to remain in the cytoplasm and be degraded by the protease system (Zhao et al., 2011). When the Hippo signaling pathway is blocked, unphosphorylated YAP1/TAZ will be transported into the nucleus to promote the transcription of downstream target genes, such as connective tissue growth factor (*CTGF*) and cysteine-rich angiogenic inducer 61 (*CYR61*) (Van Haele et al., 2019). YAP1/TAZ regulates various physiological processes, and its dysfunction has been implicated in an increasing number of human diseases (Zheng and Pan, 2019). By inhibiting YAP1 and TAZ transcriptional co-activators, the Hippo pathway regulates cell proliferation, apoptosis, and stemness in response to a variety of extracellular and intracellular signals, including cell-to-cell contact, cell polarity, mechanical cues, G-protein ligand-coupled receptors, and cellular energy status (Yu et al., 2015). In addition to the typical Hippo signaling pathway, mechanical cues can also mediate YAP1 activity independently of the Hippo signaling pathway. Thus, mechanical cues and Hippo signaling represent two parallel inputs converging on YAP1/TAZ regulation (Dupont et al., 2011; Wada et al., 2011). YAP1/TAZ can also act as on-off mechanosensing switches by sensing changes in the composition and mechanics of the extracellular matrix (ECM). The regulation of their activity has been described by a hierarchical model in which elements of Hippo pathway are controlled by focal adhesions (FAs) (Nardone et al., 2017).

Many musculoskeletal diseases are the result of long-term degeneration and often occur in the late stages of the disease (Ehrlich, 2017). As most degenerative diseases, such as OA, OP and degenerative disc disease, occur in the elderly, the global aging challenge may increase the prevalence of these diseases and their burden on society (Li and Chen, 2019). OA is the most common motor system degenerative disease in middle-aged and elderly individuals, with a survey showing that the prevalence of symptomatic OA in men and women over 60 years old was 18.0 and 9.6%, respectively. Among these individuals, 80% of patients had limited movement and 25% could not complete daily activities independently (Glyn-Jones et al., 2015). The European Society for Clinical and Economic Aspects of Osteoporosis and Osteoarthritis (ESCEO) has indicated that the cost of treating OA accounts for 1.0–2.5% of the gross domestic product of developed countries (Hiligsmann et al., 2013). In 2017, The Lancet, one of the four major medical journals, published that OA has become one the top 10 diseases leading to years lived with disability (YLDs) in China (Disease et al., 2017). OP can be divided into primary osteoporosis and secondary osteoporosis, where primary osteoporosis is more common in the clinic, and the main incidence population is the elderly and postmenopausal women (Rachner et al., 2011). OP has a high incidence worldwide (Fujiwara, 2016; Clynes et al., 2020), with the latest survey showing that the number of OP patients worldwide exceeds 200 million, and its prevalence has leaped to the seventh most common and frequently occurring disease. Studies have shown (Lin et al., 2015) that with the aging of the population, Chinese OP patients will account for more than half of the global OP patients by 2020. OP is a disease associated with aging, and as the global elderly population increases, the incidence will be greatly increased, and the associated adverse consequences will be more serious.

Thus, orthopedic degenerative diseases (OA and OP) pose great challenges to human health. Over the past decade, more and more studies have highlighted the importance of YAP1 in many diseases, including OA and OP. Because YAP1 has been less studied in degenerative disc diseases, this article reviews the role of YAP1 in orthopedic degenerative diseases (OA and OP).

## Basic Structure and Functions of YAP1

**FIGURE 1:**
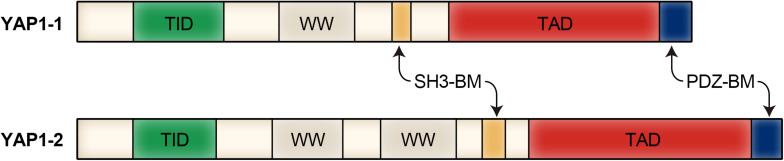
Modular structure of YAP1 protein.

YAP1 was first discovered and named by Sudol et al. (1995). Sudol et al. (1995) identified, characterized and cloned a new protein with a molecular weight of 65 Kd, it binds to the homolog Src homology domain 3 (SH3) region of the non-receptor tyrosine kinase YES. The *YAP1* gene product has two subtypes, named YAP1-1 and YAP1-2, which are distinguished by an additional 38 amino acids encoding the WW domain. The YAP1-1 subtype contains a single proline and is named Yes-associated protein (YAP65) because it binds to the proto-oncogene Yes (Sudol, 1994). Mammalian ortholog is YAP1, which has one (YAP1-1) or 2 (YAP1-2) WW domains (Sudol et al., 1995; Gaffney et al., 2012). YAP1 is primarily composed of a transcription activator binding domain (TID), a double tryptophan domain (WW domain) and a transcription activation domain (TAD). Some isomers contain an SH3 binding domain (SH3-BM) and a leucine zinc finger structure (Sudol et al., 1995). There is a proline-rich region at the N-terminus of the YAP1 protein, followed by a TEAD transcription factor interacting domain (TID) and a WW domain (Vassilev et al., 2001). The TID domain is used to recruit and bind transcriptional co-activators to regulate target gene expression together with YAP1. There is a SH3-BM behind the WW domain, followed by the TAD domain and the (PDZ domain binding motif (PDZ-BM), which primarily bind to and initiate the transcriptional expression of specific genes (Ren et al., 1993; Mohler et al., 1999). And because of the differences in regulation of transcription stage, translation level and modification level, YAP1 exhibits different functions in different types of cells. Modular structure of YAP1 protein is shown in Figure 1.

The primary function of YAP1 is to regulate stem cell self-renewal, tissue regeneration and organ size (Zhao et al., 2011; Mo et al., 2014). Under physiological conditions, YAP1 plays an important role in promoting cell proliferation, inhibiting cell apoptosis, and maintaining the multidirectional differentiation potential of stem cells, while under pathological conditions, YAP1 overexpression can easily lead to the occurrence of malignant tumors. In recent years, research on YAP1 has increased, especially with respect to tumors. YAP1 has been confirmed to be overexpressed in a variety of tumors, including gastric tumors (An et al., 2020), liver tumors (Bisso et al., 2020), ovarian cancer (Munoz-Galvan et al., 2020), and breast cancer (Song et al., 2018), suggesting that YAP1 has a role as a candidate oncogene in tumorigenesis. In addition, YAP1 also plays an important role in the migration and invasion of melanoma and osteosarcoma (Zucchini et al., 2019; Luo et al., 2020). However, the role of YAP1 in carcinogenesis is complex, with a recent study showing that the activation of YAP1 in peritumoral tissues has an inhibitory effect in cancer (Moya et al., 2019). It is noteworthy that YAP1 appears to play a tumor suppressive role in hematological cancers (Cottini et al., 2014), which contrasts with its oncogenic function in most solid tumors. YAP1 gene loci are frequently deleted in hematological cancers, and YAP1 expression or inhibition of MST1 results in inhibited growth and increased apoptosis. Therefore, additional studies are still needed to confirm the role of YAP1 in different types of tumors at different stages.

Recent studies have shown that YAP1 can regulate transcription factors critical for bone homeostasis, such as runt-related transcription factor 2 (RUNX2) (Zaidi et al., 2004), signal transducer and activator of transcription factor 3 (STAT3) (Huang et al., 2016) and β-catenin (Pan et al., 2018). And YAP1 also plays an important role in the cellular and molecular mechanisms of bone development and disease. YAP1 regulates the differentiation of chondrocyte progenitor cells (Jung et al., 2016) and interacts with several transcription factors to control gene expression in osteoblasts and chondrocytes (Deng et al., 2016). Therefore, YAP1 is essential for the proliferation and differentiation of both osteocytes and chondrocytes. In addition, YAP1/TAZ also affects the stemness development of marrow-derived mesenchymal stem cells (MSCs), which can affect the differentiation of osteoblasts (OBs), osteoclasts (OCs) and adipocytes (Wang et al., 2019). As precursor cells of chondrocytes, osteoblasts and adipocytes, MSCs are gradually becoming a potential cell source for the treatment of a variety of inflammatory, degenerative and autoimmune diseases (Su et al., 2017). The ability of MSCs to differentiate into chondrocytes provides a new approach for the repair of cartilage defects in cartilage degenerative diseases, such as OA, which has great potential for application in the repair of articular cartilage damage. When the balance between adipogenesis and osteogenesis of MSCs and the maintenance of chondrocyte phenotype is disrupted, bone and cartilage diseases such as OA and OP may occur. Therefore, YAP1 can affect the occurrence and development of orthopedic degenerative diseases (OA and OP) through multiple pathways, and this finding also provides a potential important target for the treatment of OA and OP.

## Role of YAP1 in OA

OA is the most common degenerative joint disease and is characterized by deterioration of cartilage integrity, chondrocytopenia, subchondral sclerosis, and low-grade synovial inflammation (Hawker, 2019). Chondrocytes are the only cell type present in mature cartilage, where they are dispersed in the extracellular matrix of cartilage, playing an important role in maintaining the integrity and the biological function of cartilage and having a significant role in the degeneration process of OA cartilage (Markstedt et al., 2015). Interestingly, YAP1 is involved in chondrocyte differentiation as well as phenotype maintenance and is overexpressed in OA tissues (Zhong et al., 2013a; Karystinou et al., 2015).

### YAP1 Is an Important Promoter of OA

Several studies have confirmed that YAP1 is overexpressed in OA animal model tissues, and the YAP1 content increases with the severity of OA (Nie et al., 2019; Zhang et al., 2019; Kania et al., 2020). Gong et al. (2019) reached the same conclusion in studies with an OA mouse model and human OA tissue: (1) YAP1 transcription was upregulated in knee chondrocytes of OA mice by RT-qPCR analysis; (2) YAP1 levels detected in articular chondrocytes of OA mice were upregulated by Western blot analyses; (3) and YAP1 mRNA and protein levels were increased in human chondrocytes of OA compared with normal human cartilage. This overexpression is mainly focused on the expression of the nucleus. Recent studies observed that the number of nuclear YAP1-expressing cells (52.43%) in OA cartilage was significantly higher than that detected in normal cartilage (8.39%), while the localization of cytoplasmic YAP1 was significantly lower than that observed in normal cartilage (20.02% in OA and 76.39% in normal cartilage) (Zhang et al., 2020). The level of YAP1 protein in the synovial fluid of patients with knee OA was observed to be significantly higher than that of a control group, suggesting that YAP1 protein may be an important factor in the pathogenesis of knee OA (Hwang et al., 2019). These results showed that YAP1 is significantly expressed at high levels in the OA model, suggesting that it may be involved in the development of OA and that inhibiting YAP1 expression may aid in inhibiting the development of OA.

Inflammatory mediators produced by chondrocytes play a key role in the development of OA, interleukin-1β (IL-1β) and tumor necrosis factor-α (TNF-α), which are the two most effective catabolic factors, with IL-1β having a stronger inhibitory effect on chondrogenesis than TNF-α (Heldens et al., 2012). After 24 h of treatment with IL-1β (10 ng/ml) in mouse articular chondrocytes, IL-1β was observed to significantly increase the expression of YAP1, which in turn increased the expression of matrix metalloproteinase-13 (MMP-13) and A disintegrin and metalloproteinase with thrombospondin motifs 5 (ADAMTS5) (Gong et al., 2019). ADAMTS5 and MMP-13 are well known to be important factors in cartilage degeneration associated with OA, and their expression can degrade type II collagen and proteoglycans, which leads to the excessive degradation of ECM, promoting the occurrence and development of OA (Song et al., 2007; Little et al., 2009; Scanzello, 2017). Inhibition of YAP1 expression can reverse ECM degradation and chondrocyte apoptosis induced by IL-1β (Gong et al., 2019).

However, there are a few studies showing that YAP1 plays a protective role in the progression of OA. These results of these studies suggest that YAP1 can delay the progression of OA and may allow for the treatment of OA by upregulating YAP1 (Luo et al., 2017; Deng et al., 2018; Fu et al., 2019). A recently published study by Deng et al. (2018) showed that YAP1 activation by transgene overexpression or deletion of its upstream inhibitory kinase Mst1/2 can protect the integrity of articular cartilage, while the loss of YAP1 in chondrocytes may promote cartilage destruction. It is also believed that the inflammatory reaction triggered by YAP1 by inhibiting the NF-κB signal is necessary and sufficient for delaying OA progression (Deng et al., 2018). However, in the study by Deng et al., Col2-CreER^T2^; Yap1^fl/fl^ conditional knockout mice were induced from the embryonic stage, not before the onset of OA. Furthermore, focusing only on the total expression of YAP1 in human patient samples and mouse joints without detecting the nuclear localization of YAP1 may have an impact on the final results. Therefore, YAP1 can be considered as an important promoter of OA.

### High YAP1 Expression Inhibits Chondrocyte Differentiation in OA

The pathological process of OA includes chronic inflammation and the progressive degeneration of cartilage (Vinatier et al., 2018). Studies have shown that the imbalance of chondrocyte homeostasis is associated with the development of OA (Ma et al., 2019). Therefore, it is particularly important to prevent articular cartilage degeneration and maintain the homeostasis and health of chondrocytes. In the process of chondrogenic differentiation, sex-determining region Y-type high mobility group box protein 9 SOX9) is one of the primary regulators of early chondrocyte differentiation and interacts with the transcription factors SOX5 and SOX6 to promote the proliferation and differentiation of chondrocytes, subsequently promoting the expression of chondrocyte matrix components, including aggrecan and type II collagen (Darling and Athanasiou, 2005). YAP1 has been reported to be a negative regulator of chondrogenesis, and the level of YAP1 expression has been shown to be a key element in maintaining chondrocyte function *in vitro* (Karystinou et al., 2015; Kania et al., 2020). Zhang et al. (2019) observed through qPCR and Western blotting analyses that YAP1 overexpression significantly downregulates the expression of Runx2, osteocalcin, and collagen I related to ATDC5 cell differentiation, while inhibition of YAP1 significantly enhances the expression of Runx2, osteocalcin, and collagen I. Notably, YAP1 also promotes early chondrocyte proliferation by regulating SOX6 expression and inhibiting collagen type X (COL10A1) expression by interacting with Runx2 during chondrocyte maturation, thereby inhibiting chondrocyte maturation (Deng et al., 2016; Ying et al., 2018). YAP1 negatively regulates chondrocyte differentiation by activating the β-catenin signaling pathway, and YAP1 overexpression enhances chondrocyte proliferation but inhibits chondrocyte differentiation (Yang et al., 2017). In another study of cartilage differentiation (Nie et al., 2019), dasatinib treatment was observed to significantly upregulate the serine phosphorylation of YAP1 and downregulate the protein expression of YAP1 and TAZ in a dose-dependent manner, promoting cartilage gene differentiation of MSCs by promoting the serine phosphorylation of YAP1. Goto et al. (2018) also showed that YAP1/TAZ activation in chondrogenesis can inhibit chondrocyte differentiation and maturation, leading to chondrodysplasia through a mechanism in which the activation of YAP1 or TAZ leads to decreased Sox9 expression. These findings suggest that YAP1 overexpression may play a role in the pathophysiology of OA by inhibiting chondrocyte differentiation.

### Downregulation of YAP1 Favors the Maintenance of Chondrocyte Phenotype in OA

When cartilage tissue is chronically degenerated or acutely damaged, its self-repair ability is poor, and there is little possibility of self-recovery (Buckwalter and Mankin, 1998), which ultimately leads to the occurrence of OA. The transplantation of chondrocyte biomaterials is a commonly used method to reconstruct cartilage tissue (Brittberg, 2010). However, when cultured *in vitro*, chondrocytes often fail to maintain their phenotypic stability and lose SOX9 and cartilage-specific marker expression (type II collagen and aggrecan) (Schnabel et al., 2002; Goessler et al., 2004, 2005; De Croos et al., 2006), which is not conducive to cartilage tissue reconstruction. However, promoting the maintenance of the cartilage phenotype and inducing the re-expression of cartilage-specific markers to ensure the regeneration of cartilage tissue with biochemical and mechanical functions remains a difficult challenge.

Interestingly, Zhong et al. (2013a) reported for the first time that downregulation of YAP1 expression was associated with maintenance of chondrogenic phenotype *in vitro*. In his study, high type I collagen expression in chondrocytes was observed to be accompanied by the YAP1 aggregation in the nucleus, while type II collagen expression was accompanied by the cytoplasmic localization of YAP1 (Zhong et al., 2013a). Chondrocytes are well-recognized stiffness-sensitive cells, and the establishment of an appropriate physical microenvironment of the extracellular matrix plays an important role in maintaining the chondrogenic lineage. The culturing of chondrocytes on a stiff matrix accelerates the loss of the differentiation phenotype, which appears to be well maintained on a softer matrix (Schuh et al., 2010; Allen et al., 2012). However, when YAP1/TAZ was depleted, both the proliferation rate of chondrocytes cultured on hard substrates and the expression levels of chondrogenic markers behaved as if the chondrocytes had been cultured on soft substrates (Zhong et al., 2013a). Therefore, inhibiting YAP1 in chondrocytes is beneficial to maintain the chondrocyte phenotype. In the absence of YAP1, chondrocytes can maintain their phenotype on stiff substrates. G-protein coupled receptor (GPCR) and its downstream Rho GTPase can activate YAP1/TAZ (Miller et al., 2012; Yu et al., 2012). Cell adhesion to matrix induces YAP1 localization into nucleus by activating Rho GTPase or FAK-SRC-PI3K pathway (Zhao et al., 2012). Interfering with the formation of F-actin blocked the effect of extracellular matrix on nuclear localization of YAP1. Rho GTPase and F-actin microfilaments play an important role in the regulation of YAP1/TAZ by mechanical environment. The mechanical environment includes intercellular contact, matrix adhesion, extracellular matrix hardness, basal area, etc. YAP1 has been noted to be affected by fluid shear stress in MSCs and chondrocytes, and the maintenance of the primary chondrocyte phenotype is associated with the exclusion of YAP1 from the nucleus, which may involve mechanical and cytoskeletal cues or the Hippo pathway (Zhong et al., 2013b). A recent study (Zhang et al., 2020) also observed a corresponding increase in YAP1 nuclear expression in cartilage matrix stiffness and the expression of CTGF, the most highly characteristic YAP1 target, in OA patients compared with that observed in normal patients. Finally, it was concluded that a substrate with a similar physiological hardness partially contributes to the maintenance of the chondrocyte phenotype *in vitro* through YAP1 cytoplasmic retention (Zhang et al., 2020). In summary, downregulation of YAP1 levels contributes to the maintenance of the chondrocyte phenotype, which may be associated with YAP1 mediating mechanical cues. Therefore, the phenotype of chondrocytes can be regulated by fine-tuning the expression of YAP1 to obtain the best conditions for cell implantation and facilitate the reconstruction of cartilage tissue.

**FIGURE 2:**
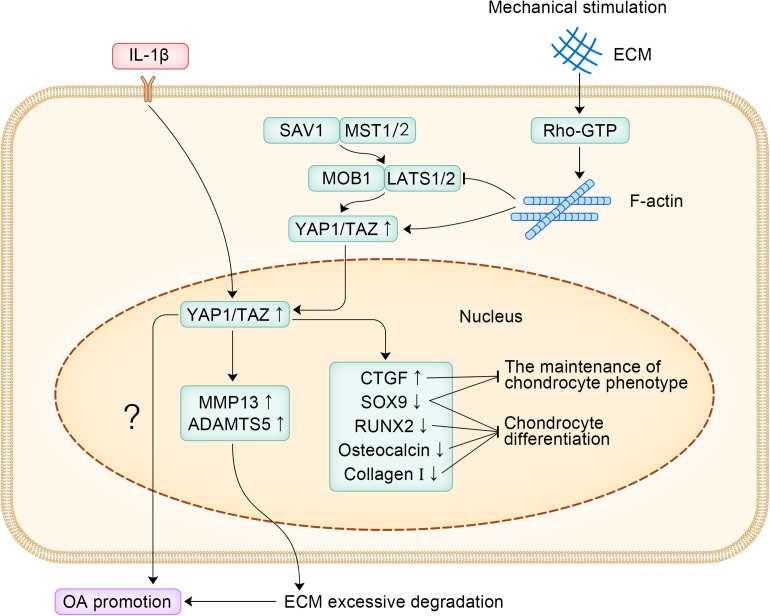
The specific signaling pathways of YAP1 promoting the occurrence and development of OA.

In summary, YAP1 is an important promoter of OA and regulates the differentiation and phenotypic maintenance of chondrocytes in OA. More research should focus on the *in vivo* studies of YAP1 in patients with OA, which will help to apply YAP1 in the clinical treatment of OA. The specific signaling pathways of YAP1 promoting the occurrence and development of OA are shown in Figure 2.

## Role of YAP1 in OP

OP is a systemic metabolic bone disease characterized by reduced bone mass and degenerative changes in bone microstructure, resulting in increased bone fragility and fracture prone (Kendler, 2011; Siris et al., 2014). OP is a major threat to the health of women and the elderly, even leading to paralysis, and it has become a chronic disease that seriously affects the health of the middle-aged and elderly population (Lin et al., 2015; Miller, 2016; Kanis et al., 2019). The Hippo signaling pathway regulates the physiological activities of osteoblasts, osteoclasts, chondrocytes and other cells through a molecular regulatory network composed of the core molecule YAP1/TAZ. Disruption of the balance of environmental or other signaling molecules causes abnormal bone metabolism and leads to bone metabolic diseases such as OP. The role of YAP1 in OP is briefly discussed below.

### Regulation of the Osteogenic Differentiation of MSCs

Adipogenesis and osteogenesis are mutually exclusive processes during MSC differentiation, and the proper balance between these processes is key to maintaining the balance of the bone marrow environment (Chen et al., 2016). Once this precisely regulated balance is disrupted, various metabolic-related diseases, including OP, may develop. Osteoinductive factors such as Runx2 have been demonstrated to inhibit adipogenesis, while peroxisome proliferator-activated receptor γ (PPARγ) stimulates adipogenesis and inhibits osteogenesis (Ge et al., 2016). Runx2 and PPARγ are subtly regulated to determine the alternating cell fate of MSCs (Fakhry et al., 2013). Other studies have also identified YAP1/TAZ as a major regulator of cell fate that promotes osteogenesis and adipogenesis in MSCs (Hong et al., 2005; Tang and Weiss, 2017). Zhong et al. (2013b) observed that with the increase of the intensity of hydrodynamic stimulation, the expression of YAP1 increased in the nucleus, which promoted the osteogenesis of MSCs, inhibited their adipogenic differentiation, and led to the dedifferentiation of chondrocytes. Another study observed that YAP1 could bind to RUNX2 and PPARγ in the nucleus, promote osteogenic differentiation of MSCs, inhibiting adipogenic differentiation and that the degree of osteogenic differentiation was positively correlated with cytoskeletal density (Pan et al., 2017). Moreover, YAP1/TAZ can reverse MMP14-induced bone loss (Tang et al., 2013).

**FIGURE 3:**
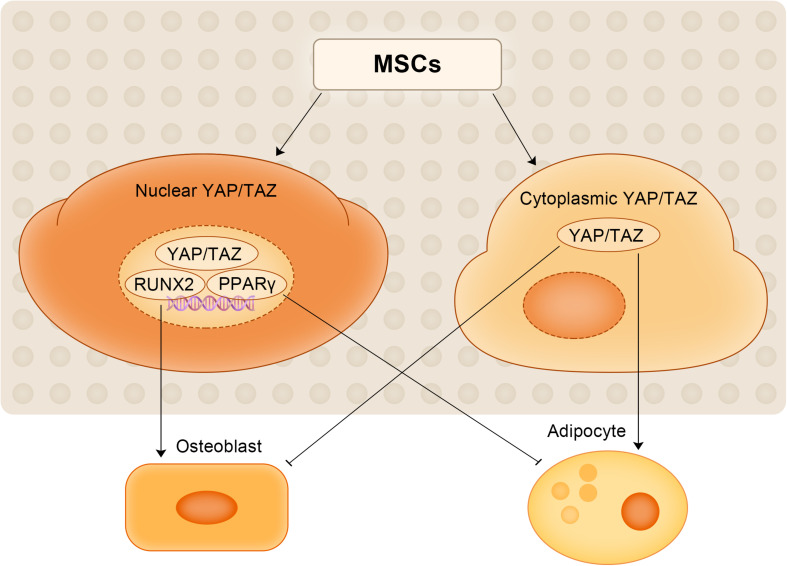
The regulatory role of YAP1/TAZ in the osteogenic and adipogenic differentiation of MSCs.

YAP1/TAZ has recently been recognized as a key regulator that promotes osteogenesis and inhibits adipogenesis through the Smad4 or β-catenin signaling pathways to maintain mouse bone homeostasis (Pan et al., 2018; Jia et al., 2019; Park et al., 2019). Pan et al. (2018) elucidated the underlying mechanism by which YAP1 regulates bone homeostasis. Through *in vivo* studies and cell culture experiments, they found that YAP1 is necessary to promote the proliferation and differentiation of mouse OB-lineage cells and to inhibit the adipogenic potential of MSCs, thereby maintaining the quality of trabecular bone. They also showed that YAP1 is required in OB-lineage cells to maintain cytoplasmic and nuclear pools of β-catenin (Pan et al., 2018). The same conclusion was reached in another study on human MSCs (Lorthongpanich et al., 2019). Studies have shown that (1) the level of YAP1 expression during human MSC differentiation is crucial for adipogenic osteogenic differentiation; (2) the ability of osteoblasts to differentiate can be enhanced by increased YAP1 levels (through drug treatment or genetic manipulation) and inhibit their differentiation into adipocytes, even if the cells are cultured in a cytokine-rich medium capable of promoting and supporting adipogenic differentiation; and (3) low YAP1 levels promotes adipogenic differentiation but inhibits osteogenic differentiation (Lorthongpanich et al., 2019). Consistent with these findings, Guo et al. (2018) also observed that in hMSCs lacking kindlin-2, YAP1 or TAZ overexpression largely restored the ability of MSCs to differentiate into osteocytes and reversed adipogenic differentiation caused by kindlin-2 deletion. Kindlin-2 is essential for regulating the osteogenic differentiation of MSCs, which increases during osteogenic differentiation and decreases during adipogenic differentiation. The regulatory role of YAP1/TAZ in the osteogenic and adipogenic differentiation of MSCs is shown in Figure 3. YAP1 regulates the balance between the adipogenic and osteogenic differentiation of MSCs and is expected to be a potential target for therapeutic intervention in OP, but additional studies are still needed to elucidate the specific mechanism by which YAP1 directs the fate of MSCs.

### Role of YAP1 in Mediating the Ability of Other Substances to Play Potential Therapeutic Roles in OP

In addition to regulating the osteogenic differentiation of MSCs, YAP1 can also mediate the ability of other substances (genes, proteins, etc.) to play a role in the treatment of OP. In the rat model of disuse osteoporosis (DOP), exosomes derived from human umbilical cord mesenchymal stem cells (HUCMSC-Exos) can reduce the apoptosis of MSCs in DOP rats through the miR-1263/Mob1/Hippo signaling pathway (Yang et al., 2020). The specific mechanism involves the miR-1263-mediated inhibition of Mob1 expression, which activates YAP1 and inhibits apoptosis. In the ovariectomized osteoporosis nude mouse model, YAP1 can mediate the therapeutic effect of melatonin toward OP, while the YAP1 inhibitor VP can attenuate the therapeutic effect of melatonin against OP (Wang et al., 2019). Another study showed that myocardin-related transcription factors (MRTFs) can regulate the balance between the adipogenic and osteogenic differentiation of MSCs, which promotes the osteogenic activity of YAP1/TAZ by maintaining the production of smooth muscle actin (SMA) in MSCs (Bian et al., 2016). Therefore, MRTF can be considered to be a novel regulator of bone homeostasis and may be a potential target for the therapeutic intervention of OP. Adequate vitamin D is important to ensure the effective prevention and treatment of OP, but vitamin D supplementation alone cannot promote calcium absorption, as it requires vitamin D receptor (VDR) to promote its biological effects (Fleet and Schoch, 2010; Shang and Sun, 2017). Recent *in vitro* studies have shown that propionic acid promotes VDR expression by activating YAP1 (Lin et al., 2020), which may be a potential pharmacological strategy for the treatment of OP.

Mechanical signals play a key role in bone growth and homeostasis (Yuan et al., 2017; Miyazaki et al., 2019). Mechanical stimulation increases bone mass by stimulating the activity and production of osteoblasts (Klein-Nulend et al., 2012; Meakin et al., 2014), and YAP1 is considered to be a mediator of mechanical signaling responses in a variety of cell types (Dupont et al., 2011; Hansen et al., 2015). Recent studies have shown that Piezo1 is a mechanically sensitive ion channel that is highly expressed in osteocytes, and its expression and activity are increased under the action of fluid shear stress (Li et al., 2019). Studies have shown that Piezo channels work through Ca2+ signaling pathways (He et al., 2018). The opening of the Piezo channels can lead to Ca2+ entry into cells, which may trigger intracellular Ca2+ signaling pathways and act as a differentiation regulator (Pathak et al., 2014). YAP1 and TAZ are necessary for WNT1 stimulation by fluids or Yoda1 (a Piezo1 agonist), demonstrating that mechanical activation of Piezo1 stimulates WNT1 expression in osteocytes, at least in part, by activating YAP1 and TAZ (Li et al., 2019). YAP1 can exert biological effects in OP by mediating the activities of other substances (genes, proteins, etc.), and other YAP1-mediated pathways, including the Piezo1-YAP1 pathway, may be a potential method to treat OP.

### Role of YAP1 in OBs and OCs Activity

The normal metabolism of bone tissue depends on the dynamic balance of OBs and OCs, which is also the basis for maintaining normal bone remodeling processes. Bone reconstruction includes the OC-mediated removal of old or damaged bone (bone resorption) and the subsequent replacement of new bone formed by OBs (bone formation) (Chen et al., 2018). OP occurs when bone formation by OBs is insufficient to combat the bone remodeling by OCs. YAP1 has been shown to play an important role in OBs and OCs.

**FIGURE 4:**
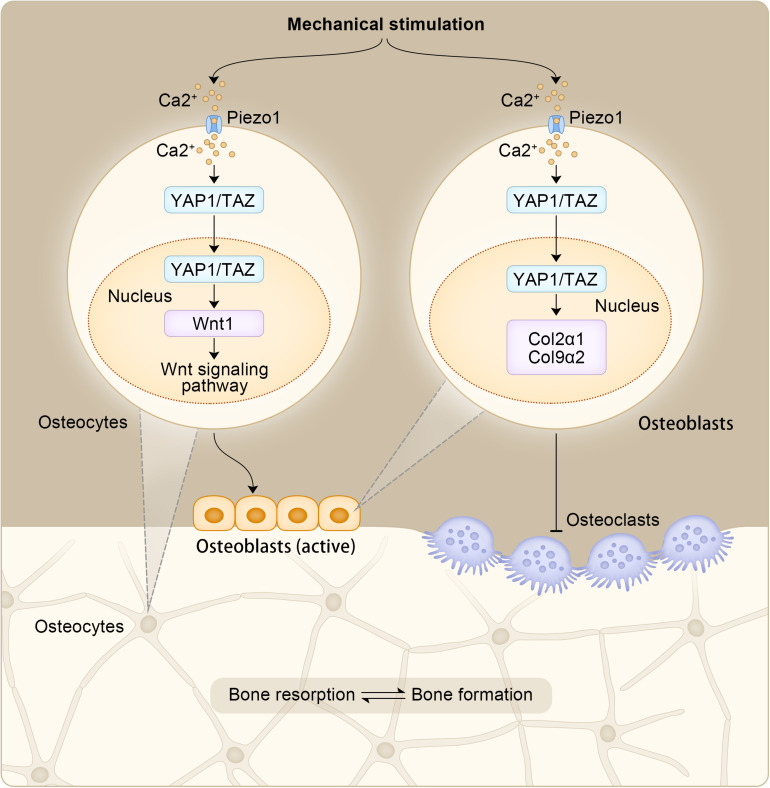
The effects of Piezo1 mediated mechanical stimulation in OB and OC.

OBs are bone-forming cells derived from mesenchymal stem cells. Kegelman et al. (2018) established a *YAP1/TAZ* gene knockout mouse model and confirmed the synergistic effect of YAP1 and TAZ at the gene level, which were shown to promote bone development by regulating the activity of OBs and the generation of OCs. However, after an individual is born, YAP1/YAZ exhibits a more complex regulatory mechanism for the OB lineage. Xiong et al. (2018) observed that YAP1/YAZ inhibits the differentiation of OB precursor cells and inhibits the transcriptional activity of RUNX2 to some extent, whereas in mature OBs and osteocytes, YAP1/YAZ promoted bone formation and inhibited bone resorption. Therefore, the regulation of OB by YAP1/TAZ is closely associated with the developmental stage of individuals and the differentiation state of cells. OC is the terminal differentiated cell of hematopoietic stem cells (HSCs) stimulated by receptor activator of nuclear factor-kappa B ligand (RANKL), which is prone to promote OP when its activity is too high (Novack and Teitelbaum, 2008). In osteoclasts, RANKL acts with cytokines in the cytoplasm to phosphorylate downstream protein kinase B (PKB/AKT) and promote osteoclastogenesis (Okamoto et al., 2017). At the same time, the effector molecule YAP1 can induce AKT phosphorylation (Jiang et al., 2017), suggesting that YAP1 may promote osteoclast formation. When YAP1 activity is inhibited by verteporfin, a small molecule drug that prevents YAP1 from binding to the TEAD domain, osteoclast formation would also be inhibited, and OC generation and bone resorption activity are inversely correlated with drug dose (Liu-Chittenden et al., 2012; Zhao et al., 2018). And as a response to mechanical stimulation, Piezo1 in osteoblasts promotes the expression of COL2α1 and COL9α2 in osteoblast cell lines through YAP1. In turn, these collagen isoforms regulate OC differentiation (Wang et al., 2020). Moreover, in osteocytes, increased Wnt1 expression resulting from Piezo1 mediated mechanical stimulation through YAP1/TAZ can activate the Wnt signaling pathway, which stimulates OBs to form new bone (Li et al., 2019). YAP1 can play a role in the occurrence and development of OP by regulating the activity of OB and promoting the generation of OC, and its role in OB and OC can also provide new ideas for the treatment of OP. The effects of Piezo1 mediated mechanical stimulation in OB and OC is shown in Figure 4.

In summary, the role of YAP1 in OP is to regulate the osteogenic differentiation of MSCs, mediate the ability of other substances (genes, proteins, etc.) to play a potential therapeutic role in OP, regulate the activity of OBs and promote the generation of OCs. More research should be focused on how to regulate the expression of the YAP1 gene and YAP1 activity to treat and prevent OP. The role of YAP1 in different cell types is shown in Table 1.

**TABLE 1:** The role of YAP1 in different cell types.

Model	Function	References
Human articular chondrocytes	The number of nuclear YAP1-expressing cells in OA cartilage is significantly higher than that detected in normal cartilage	Zhang et al., 2020
Human MSCs	YAP1 is a negative regulator of chondrogenesis in mesenchymal stem cells	Karystinou et al., 2015
Mouse MSCs	YAP1 promotes early chondrocyte proliferation by regulating SOX6 expression	Ying et al., 2018
ATDC5 cell line	YAP1 negatively regulates chondrocyte differentiation by activating the β-catenin signaling pathway	Yang et al., 2017
Mouse articular chondrocytes	The downregulation of YAP1 expression is associated with maintenance of chondrogenic phenotype *in vitro*	Zhong et al., 2013a
Mouse MSCs and articular chondrocytes	The maintenance of the primary chondrocyte phenotype is associated with the exclusion of YAP1 from the nucleus; the increased expression of YAP1 in the nucleus promotes the osteogenic effects of MSCs, inhibits their adipogenic differentiation, and leads to dedifferentiation of chondrocytes	Zhong et al., 2013b
Mouse embryonic fibroblasts and primary MSCs	YAP1/TAZ is the major regulator of cell fate that promotes osteogenesis and adipogenesis in MSCs	Hong et al., 2005
Mouse MSCs	YAP1 can bind to RUNX2 and PPARγ in the nucleus to promote osteogenic differentiation of MSCs and inhibit their adipogenic differentiation	Pan et al., 2017
Human MSCs	Low YAP1 levels promotes adipogenic differentiation but inhibits osteogenic differentiation	Lorthongpanich et al., 2019
Human MSCs	YAP1 or TAZ overexpression largely restores the ability of MSCs to differentiate into osteocytes and reverses adipogenic differentiation caused by kindlin-2 deletion.	Guo et al., 2018
Mouse MSCs	The increased expression of YAP1 in the nucleus promotes the osteogenesis of MSCs, inhibits their adipogenic differentiation, and leads to dedifferentiation of chondrocytes	Bian et al., 2016
Mouse osteoblast	The regulation of OB by YAP1/TAZ is closely associated with the developmental stage of individuals and the differentiation state of cells	Xiong et al., 2018
Mouse osteoblast	Piezo1 in osteoblasts promotes the expression of COL2α1 and COL9α2 in osteoblast cell lines through YAP1	Wang et al., 2020
Mouse osteoclast	YAP1 is an essential regulator for osteoclast differentiation and activity	Zhao et al., 2018

### Targeting YAP1 in Orthopedic Degenerative Diseases (OA and OP)

Since YAP1 is a promoter in the development of OA, and downregulation of YAP1 contributes to chondrocyte differentiation and the maintenance of the chondrocyte phenotype in OA, the downregulation of YAP1 activity may be an effective way to treat OA. Interestingly, commonly used anti-OA drugs can inhibit YAP1 expression in different ways in OA mouse models, including paracetamol, diacerein, 99Tc-MDP, DSG, O3, vitamin C, and rapamycin (Zhang et al., 2019). Verteporfin, an inhibitor of YAP1, is also a good choice, as it can disrupt the YAP1-TEAD interaction and thereby inhibit YAP1 activity (Brodowska et al., 2014). Studies have shown that the intra-articular injection of verteporfin, a selective inhibitor of YAP1, can significantly maintain cartilage homeostasis in a mouse OA model and that a sustained-release system consisting of chitosan microspheres and verteporfin has significant anti-OA effects (Zhang et al., 2020). Another YAP1 activity inhibitor, dasatinib, can also perform a similar function (Nie et al., 2019). Dasatinib can significantly inhibit YAP1 expression, promote cartilage differentiation and repair cartilage defects. Furthermore, its effect is more significant in the sustained release system composed with GelMA hydrogel. In addition to drug inhibition, YAP1 activity can also be inhibited at the gene level. SiRNA can activate RNA interference to specifically achieve mRNA degradation by complementary binding sequences with target mRNAs (Setten et al., 2019). Inhibition of *YAP1* by *YAP1* siRNA can reduce cartilage damage and chondrocyte apoptosis as well as reduce abnormal subchondral bone formation (Gong et al., 2019). Since the WW domain is essential for YAP1 to regulate biological processes such as apoptosis and autophagy (Chen et al., 2019), the activity of YAP1 may also be inhibited to some extent by deleting the WW domain.

Because YAP1 can regulate the osteogenic differentiation of MSCs and mediate the ability of a variety of substances to play a potential therapeutic role in OP, the upregulation of YAP1 activity has a positive effect on the treatment of OP. From this point of view, some YAP1 agonists may have great application prospects in the treatment of OP. The transcription factor IRF3 is a YAP1 agonist that can interact with YAP1 and TEAD4 in the nucleus to enhance their interaction and promote the nuclear translocation and activation of YAP1 (Jiao et al., 2018). Because the stimulation of glucocorticoid receptor (GR) can lead to increased protein levels and nuclear accumulation of YAP1 and promote its transcriptional activity *in vitro* and *in vivo*, glucocorticoids (GCs) can also act as YAP1 agonists (Sorrentino et al., 2017). In addition, ethacridine can also activate YAP1 and enhance TEAD-responsive reporter activity in the presence of YAP1 (Kawano et al., 2015). However, none of these YAP1 agonists have been studied in OP, and their efficacy against OP remains unknown. In addition to YAP1 agonists, YAP1 can also mediate the therapeutic effects of the genes or proteins mentioned above in OP (Bian et al., 2016; Li et al., 2019; Wang et al., 2019; Lin et al., 2020; Yang et al., 2020).

Therefore, interfering with the activity of YAP1 through drug inhibition or the modification of gene expression is a promising method for the treatment of OA, which can provide a reference for treatment strategies of OA. In OP, upregulation of YAP1 activity by YAP1 agonists or through the biological effects of other substances mediated by YAP1 also appear to be an effective therapeutic option worthy of further study. However, there are still many problems for YAP1 to become a potential disease-modifying OA drug (DMOAD), such as the selection of the optimal dose, the determination of treatment course and withdrawal indication, whether YAP1 can be combined with other DMOADs, etc.

## Conclusion

Aging alters the integrity of cells, tissues and systems, which results in a decrease in the function of the muscular system of bones and joints, this change increases the risk of developing degenerative diseases in orthopedics. However, the occurrence and development of orthopedic degenerative diseases is not an inevitable process, and if appropriate measures are taken, it is possible to restore the function of middle-aged and elderly patients. To date, there is no satisfactory drug or treatment for orthopedic degenerative diseases such as OA and OP. YAP1 has the functions of promoting cell proliferation, inhibiting cell apoptosis and maintaining the multidirectional differentiation potential of stem cells, and it has unique prospects in OA and OP. On the one hand, YAP1 is an important promoter of OA, and its high expression inhibits chondrocyte differentiation in OA, whereas its downregulation is beneficial to the maintenance of chondrocyte phenotype in OA. On the other hand, YAP1 in OP can regulate the osteogenic differentiation of MSCs and can mediate other substances (genes, proteins, etc.) to play a potential therapeutic role in OP, as well as regulate the activity of OBs and promote the generation of OCs. Therefore, the regulation of YAP1 activity through various means is expected to become a promising intervention strategy to delay the occurrence and development of orthopedic degenerative diseases. Although some progress has been made in the research of YAP1 in orthopedic degenerative diseases, many mechanisms associated with its effects remain unclear, and additional research and clinical trials are needed to elucidate the specific role of YAP1 in orthopedic degenerative diseases (such as OA and OP).

## Author Contributions

WQX and WFX conceptualized this review, decided on the content, and wrote the manuscript. KT prepared the figures. LZ and YL revised this review. All authors approved the final version of the manuscript and agreed to be accountable for all aspects of the work.

## Conflict of Interest

The authors declare that the research was conducted in the absence of any commercial or financial relationships that could be construed as a potential conflict of interest.
